# Retraction: Noncanonical TGF-β signaling leads to FBXO3-mediated degradation of ΔNp63α promoting breast cancer metastasis and poor clinical prognosis

**DOI:** 10.1371/journal.pbio.3002708

**Published:** 2024-06-27

**Authors:** The PLOS Biology Editors, Mengmeng Niu, Yajun He, Jing Xu, Liangping Ding, Tao He, Yong Yi, Mengyuan Fu, Rongtian Guo, Fengtian Li, Hu Chen, Ye-Guang Chen, Zhi-Xiong Jim Xiao

After this article [1] was published, concerns were raised about Figs 1, 2, and S4, as well as the individual-level data and raw blot data provided with this article.

Specifically:

- In Fig 1F, the Migration Flag-ΔNp63α panel and the Migration HA-TβRI Flag-ΔNp63α panel appear to partially overlap.
- In Fig 2G, there appear to be vertical discontinuities in the background of the ΔNp63α panel between lanes 6–7, 8–9, and 9–10.
- The following wound healing assay panels appear similar:
  ○ Fig S4A shGFP 0h and Fig S4D shGFP 0h
  ○ Fig S4A shGFP 48h and Fig S4D shGFP 48h
  ○ Fig S4A shFBXO3 0h panel 1, Fig S4D shFBXO3 Ctrl 0h, and Fig S4D shFBXO3 Ctrl 48h
- S1 Raw Images does not appear to present original, uncropped, and minimally adjusted images as required by the *PLOS Biology* Blot and Gel Reporting Requirements policy in place at the time this article was submitted.
- Irregularities were detected in the S1 Data file provided with the article, suggesting that the data set presented normalized data as opposed to raw underlying data.

Regarding the concerns with Fig 1, the authors stated that errors were made during the preparation of Fig 1F and Fig S4 and provided replacement panels for both experiments, as well as repeat experiment data in support of the Fig 1F results. However, the authors were unable to recover the individual image data used to quantify Fig 1G. In the absence of these data, it is unclear whether the Fig 1G results were affected by the image error in Fig 1F.

Regarding the concerns with Fig 2G and the S1 Raw Image File, the authors were unable to recover the original blots underlying this figure. The authors stated the original blots underlying Fig 1(1D, 1I), Fig 2 (2A-MCF10A, 2B-MCF10A/HaCaT, 2C, 2E-G), Fig 3B, Fig 4 (4A,4C,4G), Fig 5B, S1 Fig (S1B/C), S2 Fig (S2B/E), S3 Fig (S3A/C), and S5 Fig (S5A/C/D/E) are no longer available. The authors provided repeat experiment data, but PLOS does not consider these to be sufficient to resolve the concerns with the published article. In addition, upon editorial assessment, PLOS noticed that data provided in S1 Raw Images are neither uncropped nor minimally adjusted, as is required by *PLOS Biology*’s Blot and Gel Reporting Requirements policy. *PLOS Biology* regrets that this was not identified during the manual checks performed prior to the article’s publication.

Regarding the concerns with S1 Data, the authors clarified that the data presented in this file are the raw, unaltered data set, except for Figs 1G, 4B, 4D, 4F, 4I, 4K, 5I, 5K, S1A, S2C, S5B, S5G, S5K, S5L, and S5M, which present the normalized results. The authors provided the raw, unadjusted data set for editorial review.

Following editorial discussion of the above concerns, corresponding authors JX and MN requested retraction of the article to maintain high standards of scholarly ethics. MN, YH, JX, LD, TH, YY, MF, RG, FL, HC, and ZXJX stated that the errors do not affect the interpretations or the conclusions of the published results, and stand by the published results. YJH apologized for any inconvenience these errors may have caused.

In light of the above, which raises concerns about the reliability of the published results, the authors and the *PLOS Biology* Editors retract this article.

All authors agreed with retraction.
